# Pathogen effects on the brain: the case of SARS-CoV-2 spike protein and neuro COVID

**DOI:** 10.3389/fneur.2026.1853951

**Published:** 2026-07-13

**Authors:** Theoharis C. Theoharides, Paraskevi Papadopoulou

**Affiliations:** 1 Institute for Neuro-Immune Medicine, Nova Southeastern University, Clearwater, FL, United States; 2 Department of Immunology, Tufts University School of Medicine, Boston, MA, United States; 3 Department of Natural Sciences, School of Science and Technology, Deree-The American College of Greece, Athens, Greece

**Keywords:** brain, flavonoids, inflammation, Long-COVID, Spike protein

## Abstract

Many patients develop cognitive and neuropsychiatric issues after viral infections, but the mechanisms are not well understood. A case in point is Long-COVID syndrome, which may affect as many as 50 percent of patients after infection with severe acute respiratory syndrome coronavirus 2 (SARS-CoV-2) and is characterized by lingering physical and mental fatigue, brain fog and neuropsychiatric symptoms. Meanwhile, emerging evidence suggests the presence of inflammation around blood vessels in the brain due to the Spike protein remaining in “reservoirs” especially in the meninges that contain great numbers of the unique tissue immune cells, mast cells (MCs). In fact, Spike protein has been reported to stimulate MCs and microglia to release pro-inflammatory, neurotoxic and vasoactive mediators leading to Long-COVID and other neurodegenerative disorders. Thus, it is of great urgency to gain insight into how the Spike protein and neuroinflammatory molecules contribute to Long-COVID and how to regulate this response.

## 1 Introduction

As many as 50 percent of patients infected with severe acute respiratory syndrome coronavirus-2 (SARS-CoV-2) develop post-acute sequalae of SARS-CoV-2 (PASC) within a few months after the initial infection (1–4), also referred to as “Post-acute COVID” (2), “Long-COVID” or “Neuro-COVID” (2). Long-COVID is characterized by persistent fatigue, which is independent of the initial severity of the infection (5, 6). In addition, many patients with Long-COVID may have persistent symptomatology even as long as seven months post-acute infection (7, 8), which appears to be more common with increasing age and female sex (3). Long-COVID patients suffer from a spectrum of neurological disorders including headache, anosmia, encephalopathy, peripheral neuropathy, and cerebrovascular events (9–17); neurodegenerative changes resembling early Alzheimer's and Parkinson's pathology (12, 18, 19); psychiatric disorders including depression, anxiety, PTSD, and sleep disturbances (20–26); and cognitive impairment encompassing brain fog (2, 6, 8, 27–32), memory deficits, executive dysfunction (21–26, 33–35) and peripheral neuropathy (36). These manifestations arise through multiple mechanisms including direct Spike-protein-driven neuroinflammation, microglial and MC activation, Blood Brain Barrier (BBB) disruption, HPA-axis dysregulation, hypoxia, and immune-mediated injury. Over 90 percent of patients who were initially hospitalized for COVID-19 infection and had neurological symptoms, including encephalopathy, acute confusion, agitation, corticospinal tract signs, and stroke, consistent with data from Helms et al. (9) and Taquet et al. (26), experienced significantly worse outcomes 6-months post-acute infection (37). The duration of Long-COVID symptoms is not known, but recent data indicate that it may depend on antigen persistence (38), and sustained specific immune responses to SARS-CoV-2 (39).

Table 1 summarizes Long-COVID associated disorders, their approximate prevalence, and proposed mechanisms. The mechanistic categories addressed include: (i) direct neurotropism of SARS-CoV-2 or its Spike protein; (ii) neuroinflammation driven by microglial and MC activation; (iii) HPA-axis dysregulation and CRH-mediated effects; (iv) BBB disruption; (v) hypoxia and cerebrovascular injury; and (vi) immune-mediated and autoimmune injury.

**Table 1:** Neurological, neurodegenerative, psychiatric, and cognitive manifestations of Long-COVID: Prevalence and proposed mechanisms.

Category/Sources	Disorder	Approximate prevalence	Proposed mechanism(s)
Neurological (15, 37, 340–342)	Headache	~47%	Trigeminovascular activation; cytokine-mediated meningeal irritation; cerebral vasospasm
Neurological (15, 37, 340–342)	Anosmia/Hyposmia	~11–20%	Olfactory neuroepithelial inflammation; ACE2–mediated olfactory neuron damage
Neurological (15, 37, 340–342)	Peripheral Neuropathy	~5–10%	Immune–mediated axonal injury; complement activation; small–fiber neuropathy
Neurological (15, 37, 340–342)	Stroke/Cerebrovascular	~1–2%	Hypercoagulability; endothelial dysfunction; paradoxical embolism
Neurological (15, 37, 340–342)	Encephalopathy/Delirium	~5–8%	Hypoxia; cytokine storm; BBB disruption; direct viral neurotropism
Neurodegen. (12, 36, 65, 341)	Accelerated Alzheimer's–like changes	Emerging data	Neuroinflammation triggering tau hyperphosphorylation; amyloid–β accumulation; microglial overactivation
Neurodegen. (12, 36, 65, 341)	Parkinsonism/Dopaminergic dysfunction	Case reports/small cohorts	Basal ganglia inflammation; TLR4–mediated nigrostriatal damage; α-synuclein aggregation
Neurodegen. (12, 36, 65, 341)	Chronic microglial activation	Histopathological findings	Sustained NLRP3 inflammasome activation; MC–microglia crosstalk; Spike protein persistence
Psychiatric (34, 343–345)	Depression	~12–23%	HPA–axis dysregulation; reduced serotonergic transmission; cytokine–induced tryptophan depletion
Psychiatric (34, 343–345)	Anxiety/GAD	~15–22%	Amygdala hyperactivation; neuroinflammation; sustained immune activation
Psychiatric (34, 343–345)	PTSD	~12–16%	Trauma of severe illness/ICU stay; hippocampal atrophy; CRH–mediated fear conditioning
Psychiatric (34, 343–345)	Sleep disturbances	~25–30%	Cytokine–driven circadian disruption; locus coeruleus dysfunction; serotonergic dysregulation
Cognitive (30, 32, 83, 345–347)	Brain fog/Executive dysfunction	~20–30%	Microglial hyperactivation; reduced cerebral perfusion; PAF–mediated platelet–neurovascular injury
Cognitive (30, 32, 83, 345–347)	Memory impairment	~19–28%	Hippocampal neuroinflammation; disrupted synaptic plasticity; oxidative stress–mediated neuronal loss
Cognitive (30, 32, 83, 345–347)	Attention deficits	~20–25%	Prefrontal cortex hypoperfusion; thalamo–cortical network disruption; chronic fatigue–related inhibition

### 1.1 Long-COVID epidemiology across SARS-CoV-2 variants

The epidemiology of Long-COVID varies across SARS-CoV-2 variants of concern. Data from large cohort studies indicate that infections with the ancestral WT strain were associated with the highest prevalence of persistent symptoms approximately 30–50% for the ancestral WT strain in symptomatic/hospitalized cohorts (40, 41), with intermediate rates for Alpha and Delta (20–45%) (40–42) and lower overall prevalence for Omicron (~23–29%), though neurological manifestations appear relatively more prominent in Omicron (41, 43–46). The differential Spike protein mutations across variants, most notably the over 30 receptor-binding domain (RBD) mutations in Omicron BA.1 relative to the ancestral strain, may modulate Angiotensin Converting Enzyme 2 (ACE2) binding affinity and activation potential of toll-like receptors (TLR) (47). Nevertheless, key functional domains of the Spike protein relevant to neuroimmune signaling appear broadly conserved across variants, suggesting that the mechanisms described in this review apply across the pandemic era, albeit with variant-specific nuances that warrant dedicated future investigation.

### 1.2 Long-COVID pathogenesis is unknown

The mechanism by which Long-COVID occurs has yet to be fully elucidated. It is well understood that SARS-CoV-2 infects cells by first binding to its surface receptor, ACE2, via its corona Spike, subunit 1 (S1) protein (48). Infection leads to a complex immune response (49) that involves the release of a “storm” (50, 51) of pro-inflammatory cytokines (49–55), especially IL-1b (56, 57) and IL-6 (58–61), increased levels of which have been detected in the cerebral spinal fluid (CSF) of patients with COVID-19 infection (62, 63). This has been demonstrated to contribute to the development of acute and persistent symptomatology (50, 64). Still, to date, the effect of SARS-CoV-2 on the brain is not well understood.

### 1.3 Importance of microglia activation in COVID-19

Microglia are central nervous system (CNS)-resident mononuclear phagocytes that constitute between five and 10 percent of total brain cells and seed the brain early in development (65–67). Microglia have important functions in the CNS (68), especially with respect to neuroinflammation (68, 69) and neurodegenerative (65, 70, 71) diseases. They are the principal immune effectors of the CNS and play essential homeostatic roles including synaptic pruning, surveillance for damage-associated molecular patterns (DAMPs), and regulation of neuroinflammation. Microglia express TLRs (72) activated by damage associated molecular patterns (DAMPs and have been implicated in COVID-19 (73, 74). Microglia express receptors for corticotropin-releasing hormone (CRH), secreted under stress (75), especially associated with COVID-19 infection (18) that can also affect the hypothalamic-pituitary-adrenal (HPA) axis (76), further affecting the emotional state of individuals affected by the corona virus (77, 78).

Whether SARS-CoV-2 directly infects microglia remains debated. While some in vitro studies using microglial cell lines (HMC3) demonstrated viral entry and M1-like pro-inflammatory activation followed by apoptosis (79), analyses of human post-mortem brain tissue and more physiologically relevant experimental models have not consistently confirmed productive microglial infection (80). Instead, the evidence from post-mortem analyses of COVID-19 brains have shown extensive microglial activation, neuroinflammation and microglial nodules in the medulla and cerebellum. In the K18-hACE2 transgenic mouse model, intranasal SARS-CoV-2 infection induced vasculitis, gliosis and vascular inflammatory changes without necessarily requiring direct microglial infection (81). In this review, we therefore emphasize indirect Spike-protein-driven activation of microglia, via TLR4 signaling, BBB-disruption-mediated cytokine entry, and inflammatory, neurotoxic and vasoactive mediators derived from the unique tissue immune cells, the mast cells (MCs), as the primary neuroinflammatory mechanisms involved in Neuro-COVID.

### 1.4 Microglia-induced neuroinflammation

One study demonstrated that SARS-CoV-2 Spike (S1) protein elicited a robust NF-κB/NLRP3 inflammasome-mediated pro-inflammatory response in BV-2 microglial cells (82). In addition, post-mortem analysis of brains obtained from deceased patients with COVID-19 showed extensive microglial activation and neuroinflammation associated with brain pathology (83–85). A recent study using transgenic mice injected by SARS-CoV-2 slowed vasculitis gliosis, and vascular inflammatory changes in the brain (81). SARS-CoV-2 neurotropism may trigger or exacerbate neuropsychiatric disorders (86) since microglia-induced neuroinflammation is a risk factor for the development of major depressive disorder (87). Increasing evidence indicates the involvement of neuro-inflammation (88–90), which may damage brain blood vessels, (91, 92) and brain cells (88, 93, 94), possibly via activation of microglia (95, 96). As such, Long-COVID could be considered a state of “brain autoimmunity” (97).

An important and underappreciated feature of neuro-COVID pathophysiology is the reciprocal crosstalk between microglia and MCs. This feedforward MC-microglia loop has significant neurological consequences, including sustained neuroinflammation, BBB disruption, hippocampal synaptic dysfunction leading to brain fog and memory impairment, and potentially accelerated neurodegeneration, all hallmarks of the Long-COVID neuropsychiatric syndrome.

Activation of the HPA axis (itself dysregulated in COVID-19) leads to CRH release, which stimulates perivascular MCs to secrete vasoactive and pro-inflammatory mediators including IL-1β, IL-6, TNF-α, and tryptase. These mediators in turn activate microglia and increase BBB permeability, further allowing peripheral immune signals to enter the CNS (98).

This feedforward loop in which MC activation amplifies microglial reactivity and vice versa, is proposed as a central pathological axis in Long-COVID neuroinflammation and is elaborated in the sections below (99).

### 1.5 Inflammation, microglia and mast cells

**Figure 1:**
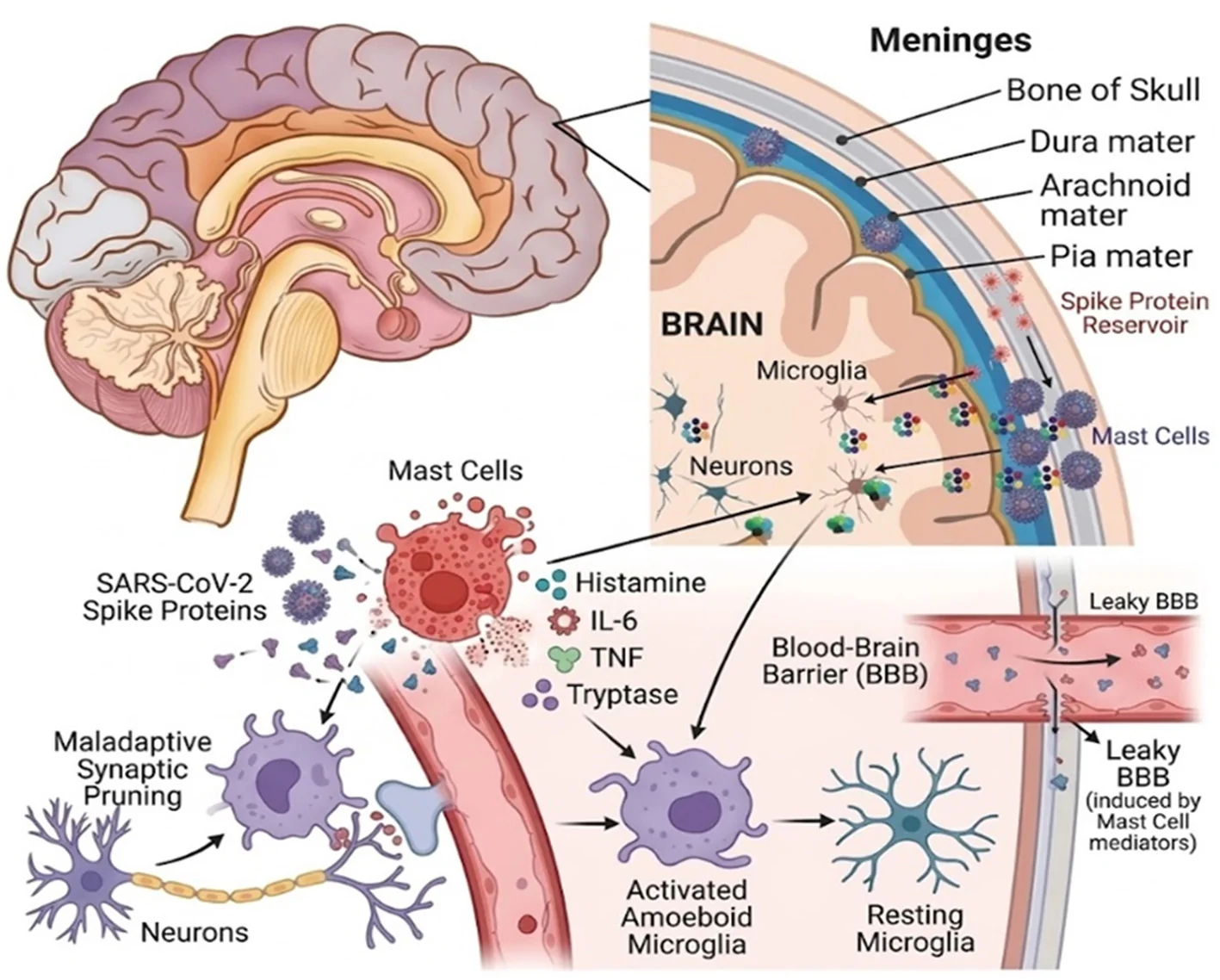
Diagrammatic representation of how SARS-CoV-2 Spike protein can exist in a meningeal “reservoir” where it can stimulate meningeal mast cells directly leading to subsequent activation of microglia and disruption of the BBB permitting the Spike protein to cross the BBB and activate mast cells and microglia leading to neurovascular inflammation. Designed using Canva and Gemini-AI.

Microglia have been shown to play a key role in brain injury (100), as well as the development of neuroinflammatory (89, 101–103) and neurodegenerative diseases (65, 68–71, 89, 101, 104–107). Microglia can interact with MCs (108, 109) in neuroinflammatory (110–113) and neurodegenerative diseases (86, 110, 114–116). MCs are ubiquitous in the body (117) and are critical for allergic diseases (118), including mastocytosis (108) and other MC activation disorders (119). MCs are present in the autonomic nervous system (ANS) (120) and the CNS perivascularly (121, 122), especially in the meninges (123, 124), amygdala, hippocampus, thalamus (125–127) and hypothalamus (68, 123, 127). In addition to allergies, MCs are also involved in inflammatory processes (113, 128), neuroinflammation (111, 129) and the regulation of the BBB) (121, 130, 131) (Figure 1). Other than allergens acting via immunoglobulin E (IgE) bound to surface receptors (FcεRI), MC are stimulated by non-allergic agents (108, 118, 132, 133), additional neuropeptides, including neurotensin (NT), a CRH co-activator present in perivascular MCs, further amplify this response (134, 135), such as CRH (136) and neurotensin (NT) (137), which have pro-inflammatory properties (138–141). Activation of MCs (142, 143) and microglia (144), especially in the hypothalamus (145) and the hippocampus (146), could lead to cognitive dysfunction (147), which is also seen in patients with MC disorders (148–150).

Table 2, a compilation from the previous paragraph references, depicts the brain-related actions of MC mediators and the mechanism of these actions and what neurological consequences they have.

**Table 2:** Brain-related actions of mast cell mediators: Mechanisms and neurological consequences.

Brain-related action	Key mediator(s)	Mechanism of action	Neurological consequences
Activation of microglia, astrocytes & neurons	Tryptase, chymase, IL-1β, IL-33, TNF-α	Tryptase activates PAR-2 on microglia; IL-1β/IL-33 engage IL-1R/ST2 receptors triggering NF-κB and NLRP3 inflammasome activation	Sustained neuroinflammation; synaptic dysfunction; microglial M1 polarization contributing to brain fog and cognitive impairment
Disruption of the BBB	Histamine, VEGF, PAF, tryptase, heparanase	Histamine acts on H1/H2 receptors on endothelial cells reducing tight junction expression (ZO-1, occludin); VEGF increases vascular permeability; tryptase degrades basement membrane components	Increased CNS entry of peripheral cytokines, immune cells, and Spike protein; cerebral edema; enhanced neuroinvasion
Secretion of proinflammatory, neurotoxic & vasoactive mediators	IL-6, TNF-α, PGD2, LTC4, ROS	IgE- and non-IgE-mediated degranulation releases preformed and newly synthesized mediators via PKC/MAPK/Ca^2+^ signaling cascades; Spike protein activates via TLR4/ACE2	Neuronal oxidative stress and cytotoxicity; vasospasm; amplification of the neuroinflammatory cascade; contributes to fatigue and pain sensitization
Cognitive decline	Histamine, tryptase, IL-6, CRH	Hippocampal and prefrontal MC-microglial activation disrupts LTP and synaptic plasticity; histamine alters neurotransmitter balance; CRH dysregulates HPA axis impairing memory consolidation	Brain fog, memory deficits, impaired executive function, and attention deficits characteristic of Long-COVID
Regulation of the HPA axis & stress response	Histamine, IL-6, CRH (released and stimulated)	MCs express CRH receptors (CRH-R1) and release CRH themselves; IL-6 and histamine act on the hypothalamus to stimulate ACTH and cortisol release	HPA axis dysregulation; mood disturbances; anxiety; depression; PTSD-like symptoms; impaired stress resilience in Long-COVID
Extracellular matrix disruption	MMP-9, heparanase, chymase	Tryptase and chymase cleave extracellular matrix (ECM) proteins; MMP-9 degrades collagen IV and laminin in the perivascular basement membrane	Perivascular neuroinflammation; impaired neurovascular coupling; glymphatic dysfunction; facilitation of immune cell infiltration
Neurodegeneration	ROS, MMP-9, TNF-α, neurotoxic proteases	Sustained oxidative stress and protease activity cause neuronal apoptosis; TNF-α induces excitotoxicity via glutamate dysregulation; chronic microglial activation promotes tau hyperphosphorylation and α-synuclein aggregation	Progressive neuronal loss; potential acceleration of Alzheimer‘s- and Parkinson's-like pathology in Long-COVID patients
Early responders in brain injury	Preformed granule contents: histamine, tryptase, chymase, heparin	Rapid degranulation (< 30 seconds) upon DAMPs/PAMPs detection; MCs act as sentinel cells amplifying innate immune responses before microglial activation	Initiates and amplifies acute neuroinflammatory cascades; worsens secondary brain injury; sets the stage for chronic neuroinflammation in Long-COVID

BBB, blood-brain barrier; CRH, corticotropin-releasing hormone; DAMPs, damage-associated molecular patterns; ECM, extracellular matrix; HPA, hypothalamic-pituitary-adrenal; IL, interleukin; LTC4, leukotriene C4; LTP, long-term potentiation; MAPK, mitogen-activated protein kinase; MMP-9, matrix metalloproteinase-9; NF-κB, nuclear factor kappa B; NLRP3, NLR family pyrin domain-containing protein 3; PAF, platelet-activating factor; PAR-2, protease-activated receptor 2; PGD2, prostaglandin D2; PKC, protein kinase C; ROS, reactive oxygen species; TNF-α, tumor necrosis factor alpha; VEGF, vascular endothelial growth factor.

Functional interactions have also been reported between MCs and neurons (123, 151, 152), often positive for CRH (123, 153). MCs can activate the HPA axis (136, 154, 155) via release of histamine (156), IL-6 (157–159) and CRH (160). We reported that serum levels of CRH were elevated in children with autism spectrum disorder (ASD) (161). CRH has been documented to play a critical role in neuroimmune responses in the skin (162) via activation of MCs (134). CRH augmented IgE-stimulated human MC release of vascular endothelial growth factor (VEGF) (163, 164). We had reported that children born to mothers with mastocytosis and with atopic diseases (97) had a higher risk of developing ASD. We then advanced the premise that focal inflammation in the amygdala (52, 53), which regulates behavior and fear (145, 165–167), could result from activation of microglia with release of inflammatory, neurotoxic, and tissue-disrupting molecules (166). We showed that psychological stress (78, 168) increased the reactivity of MCs (78), leading to increased BBB permeability (98, 142, 169). MC-derived mediators, such as cytokines (170), increased BBB permeability (98, 171–173) via CRH stimulating MCs (171, 174), an effect that was absent in MC-knockout mice (175). In addition, NT increased skin vascular permeability via a CRH-dependent process (176) and NT further induced the expression of CRH receptor-1 in human MCs (177), in which CRH stimulated the secretion of VEGF (178). We further reported that NT can activate human microglia to secrete pro-inflammatory molecules and increase expression of receptors for NT. We reported, utilizing RNAseq profiling on postmortem brain samples from male children with ASD and controls, that the hippocampus of brains from children with ASD had decreased expression of genes related to synaptic connectivity and increased expression of proinflammatory genes, especially matrix metalloproteinase-9 (MMP-9) (179).

Neuroimmune transcriptome analysis in post-mortem brains revealed altered expression of immune-related genes in ASD (180), supporting deficits in neuronal connectivity. Similar pathological processes may explain some of the neuropsychiatric sequllae of Long-COVID. Microglial activation has also been associated with aging and cognitive decline (181). MC-derived mediators (182), including histamine (183) and tryptase (184), can activate microglia in the brains of children with ASD (185–188) and other neuroinflammatory disorders.

### 1.6 Significance of SARS-CoV-2 Spike protein

**Figure 2:**
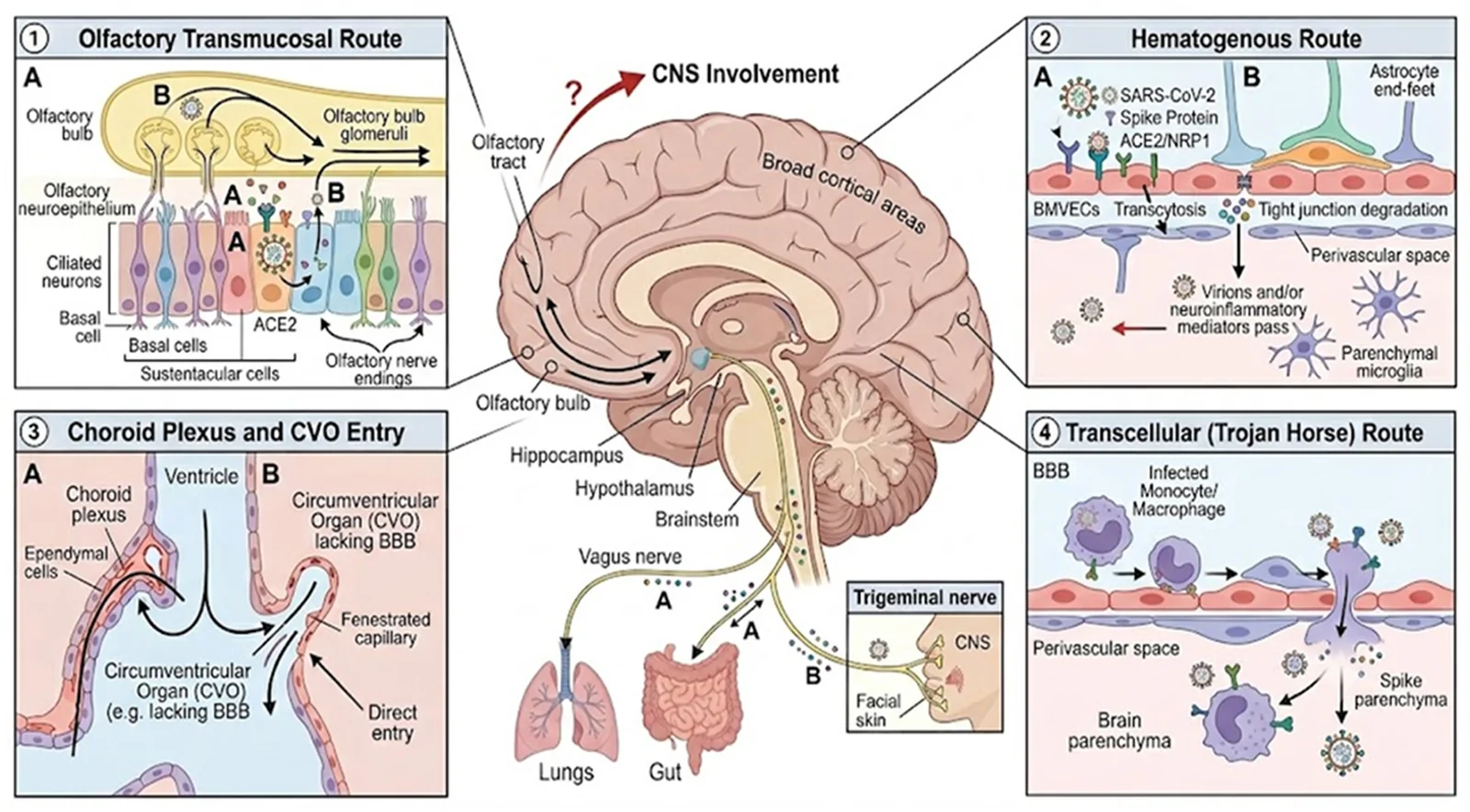
Proposed routes of SARS-CoV-2 and Spike protein CNS entry.

The neurologic effects of COVID-19 may be attributed to SARS-CoV-2 entering the brain, but the pathways of such neurotropism are still unclear (189, 190). Figure 2 illustrates the possible pathways of entrance in the brain. The S1 protein is trimeric and catalyzes fusion between the viral and host cell membrane. This “prefusion” trimer has three receptor-binding domains (RBD), while the post-fusion structure expresses N-linked glycans that may serve to protect against immune responses (191). Previous studies demonstrated that the virus crosses or damages the BBB (90), via a transcellular route, accompanied by basement membrane disruption, without significant tight junction alteration (192), similarly shown in an animal model of K18-hACE2 where transgenic mice were infected with SARS-CoV-2 (81). The virus was also detected in human cortical neurons (193). In another study, a fragment specific to SARS-CoV-2 was amplified from cultures of a brain specimen from a deceased patient with COVID-19 and associated pathology showed neuronal necrosis and glial cell hyperplasia (194). Alternatively, the virus could enter from the nose by crossing the neural-mucosal interface of the olfactory nerve (195) and then enter the brain via the olfactory nerve tract (196). Viral entry into the brain via gustatory-olfactory trigeminal pathway eventually compromising the BBB was recently reported in deer mice infected with SARS-CoV-2 (197). It is interesting that single-cell RNA sequencing (RNA-Seq) showed that ACE2 was not expressed by olfactory sensory or bulb neurons, but instead was expressed by olfactory epithelium and pericytes (198). In the autopsy report of an infant who died with COVID-19, there was evidence of cortical atrophy and severe neuronal loss, findings restricted to capillaries of the choroid plexus (199). Furthermore, another study did not document any molecular traces of SARS-CoV-2 in the brains of deceased patients with COVID-19, but detected choroid plexus perturbations associated with pathologic morphological changes in the microglia (200). Such pathology may be explained by the expression of the ACE2 receptor by human glial cells and neurons (201).

### 1.7 Circulating spike protein persistence in Long-COVID

A critical and rapidly growing body of evidence supports the persistence of SARS-CoV-2 antigens, including Spike protein fragments, in the blood and tissues of Long-COVID patients long after resolution of the acute infection. Swank et al., 2023 found that full-length Spike antigen was detectable in plasma from approximately 60% of PASC patients at any post-recovery time point, with measurements extending up to 17 months, while remaining undetectable in plasma from acute COVID-19 patients in the first week of illness (202). The S1 subunit was found in approximately 20% of PASC patients. Peluso et al. (203) confirmed antigen persistence over a 14-month follow-up in a well-characterized longitudinal cohort, finding that plasma Spike antigen levels correlated with persistent immune activation markers. This evidence of circulating Spike protein provides the biological basis for the model presented in this review: if Spike protein persists in plasma, it has the opportunity to engage circulating monocytes and tissue-resident macrophages and MCs; to encounter the BBB and promote its disruption; and to enter the perivascular and meningeal space, where it may stimulate MCs and microglia to perpetuate neuroinflammation.

The comprehensive reservoir framework by Proal et al. (204) synthesizes evidence for SARS-CoV-2 RNA and protein persistence across multiple tissue compartments including lymph nodes, gut, and potentially neural tissues. The presence of SARS-CoV-2 Spike protein “reservoirs” have been identified (204–207), especially in the gut (208) and the meninges (209). It has been proposed, but not yet directly demonstrated, that the Spike protein may be taken up and stored in macrophages and MC macrophages (210) and MCs (211), which are plentiful especially in the meninges (Figure 1). This hypothesis is biologically plausible given the known phagocytic capacity of these cells, their long tissue residency, and established roles in antigen storage; however, it currently rests on indirect evidence and requires direct experimental confirmation using Spike-specific detection assays in sorted cell populations. There is precedent for MCs storing viral particles as it had been reported for HIV (212–214). These findings collectively justify the priority of identifying Spike protein persistence and its downstream neuroimmune consequences as a central research direction in Long-COVID (202–204, 207). Potential therapeutic strategies to eliminate Spike reservoir have been reviewed (207).

### 1.8 Primary experimental evidence for Spike-MC activation

Importantly, activated MCs do not act in isolation; through release of proteases such as tryptase and chymase, and cytokines including IL-33, TNF-α, and IL-1β, they can directly activate microglia, creating a feedforward neuroinflammatory loop that amplifies brain injury in Long-COVID. Primary experimental evidence supports the capacity of SARS-CoV-2 Spike protein to directly activate MCs. Tsilioni and Theoharides (215) demonstrated that recombinant full-length Spike protein (but not the isolated RBD) stimulates human cultured MCs to release interleukin-1β, chymase, and tryptase through TLR4- and ACE2-dependent mechanisms, respectively, an effect augmented by IL-33 co-stimulation. This finding is corroborated by in vivo and ex vivo studies: (216). Tan et al. (217) demonstrated MC activation and degranulation in lung tissue from SARS-CoV-2-infected mice and humans, correlating with disease severity; Wu et al. (218) showed that SARS-CoV-2 Spike-triggered MC degranulation induces alveolar epithelial inflammation (218); and Zhang et al. (219) elucidated the intracellular mechanism of Spike-driven mast cell degranulation via a Src/PI3K/AKT/Ca^2+^ signaling cascade. Importantly, most of this evidence derives from peripheral or in vitro models; direct evidence for Spike-driven MC activation specifically within the CNS meningeal compartment remains to be established in appropriate experimental models (109, 220).

### 1.9 Platelets and PAF

Previous publications have reported that COVID-19 is characterized by perivascular inflammation in the brain (91, 221–223), microthrombi (224, 225) decreased platelet counts, as well as by their hyperactivation since P-selectin expression and integrin αIIbβ3 activation are both increased in most cases. Plasma levels of platelet-derived growth factor (PDGF) are also elevated and washed platelet aggregation induced by ADP, thrombin and collagen was increased mainly in critically ill COVID-19 patients (226–229). Moreover, Spike protein was reported to induce P-selectin expression and integrin αIIbβ3 activation but there are conflicting results regarding its ability to induce platelet aggregation in human washed platelets in the absence of any other agonist (230, 231). Interestingly, both platelets (91, 232) and PAF (91, 233) have also been implicated in inflammation.

It was reported that platelet rich plasma (PRP) from vaccinated individuals, exhibited *ex vivo* lower EC_50_ values in response to PAF, ADP and collagen. Platelet incubation with the Spike protein alone resulted in augmentation of *in vitro* PAF-induced platelet aggregation (223). In addition, the authors showed that the Spike protein induced a 2-fold increase in intracellular PAF production. The ability of the Spike protein to induce the production of the potent mediator, PAF, along with other proinflammatory cytokines may in part mediate the effects of SARS-CoV-2 and potentially contribute to LONG-COVID syndrome (234). PAF is linked to thrombosis, inflammation, and atherosclerosis.

One study (235) examined 47 patients from the COVMENT trial, assessing cognitive performance via the Montreal Cognitive Assessment (MoCA), brain glucose metabolism through fluoro-2-deoxy-d-glucose (FDG) PET-CT, and various inflammatory markers. Results indicated a significant correlation between lower MoCA abstraction scores and diminished FDG uptake in brain regions crucial for abstract reasoning. Among inflammatory markers, only the platelet-to-lymphocyte ratio (PLR) showed significant ties to brain metabolism and cognitive performance, with lower values linked to greater neurometabolic impairment or brain fog. The findings suggest a connection between chronic immune dysregulation and cognitive dysfunction in post-COVID-19 patients.

Increasing evidence suggests platelet-derived biotherapies are gaining attention for treating complex neurological disorders through multimodal interventions (236). PAF inhibitors, both natural and synthetic, can be useful in preventing and treating neurodevelopmental disorders by targeting the PAF signaling pathway. These inhibitors may provide significant therapeutic benefits, including anti-inflammatory effects and slowing disease progression, alongside a review of current therapeutic strategies and future outlooks (237). An in vitro study reported the effects of the flavonoid quercetin on platelet inactivation and thrombosis prevention (238) and NF-κB identified as a key target.

Wine and olive oil's protective effects against atherosclerosis are primarily due to phenolic compounds like resveratrol (RSV/RES), and tyrosol, which inhibit PAF biosynthesis in stimulated monocytes. IL-1β activates PAF biosynthesis through various signaling pathways, with phospholipase C-β (PLC-β) as a crucial enzyme (239, 240). One study examined the impact of resveratrol, tyrosol, and their derivatives on unstimulated U937 cells and the intracellular pathways influencing PAF biosynthesis. While tyrosol and its derivatives showed minimal effects, resveratrol (50 and 100 μM) and its methoxy derivative (5–20 μM) reduced PAF biosynthetic enzyme activity by 20–43% after 24 hs. Conversely, low resveratrol concentrations (10 μM) and high methoxy derivative concentration (50 μM) increased lyso-PAF acetyltransferase activity (28–45%) after 30 min via p38-MAPK action. RSV/ RES, is a polyphenol primarily from red grapes, and has garnered attention for its pharmacological properties, including antioxidant and anti-inflammatory effects. Key intracellular pathways such as NF-κB, JAK/STAT, MAPK/ERK PI3K/Akt, and Nrf2/Keap1 demonstrate RSV/RES's potential in modulating immune responses, reducing oxidative stress, and promoting autophagy (241). Despite limitations in pharmacokinetics, RSV/RES's ability to penetrate the BBB encourages ongoing research to improve its CNS delivery. Challenges related to RSV/RES, such as low bioavailability and dosing variability, hinder clinical application. Further well-structured clinical trials are necessary to assess its efficacy and safety in human studies, emphasizing its potential in neuroprotective strategies for neurological conditions (241).

### 1.10 Lack of effective treatments

**Figure 3:**
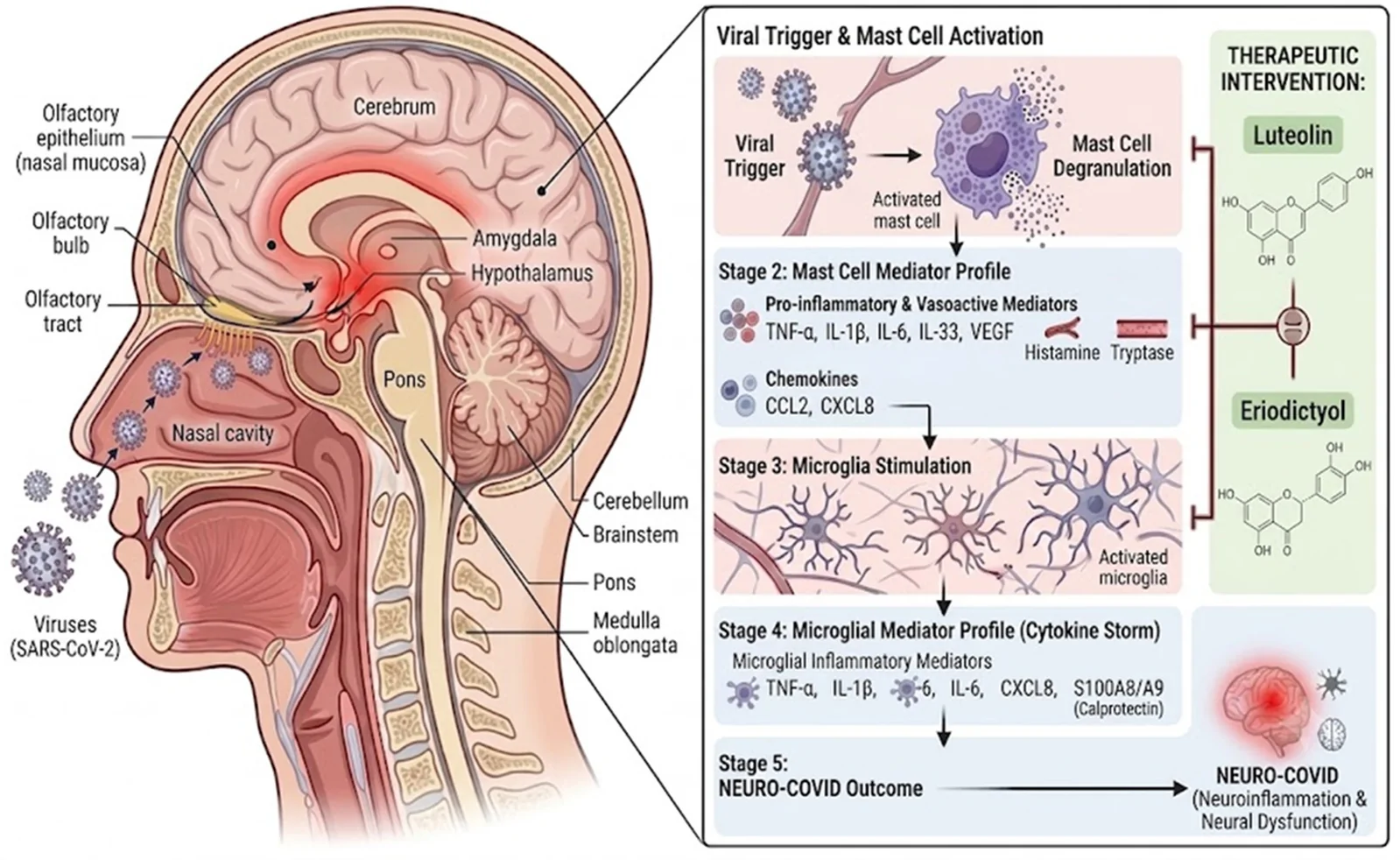
Schematic diagram showing the proposed beneficial effects of eriodictyol and luteolin. Long-COVID (Neuro-COVID) can activate mast cells and several inflammatory mediators released from activated mast cells can activate microglia and other brain cells to release inflammatory and neurotoxic mediators that can cause neuroinflammation, and neurodegeneration contributing to the development of neuro-COVID. Eriodictyol could inhibit Long-COVID-associated inflammatory mediator release from activated mast cells and microglia. Designed using Canva and Gemini-AI.

To date, there are no effective drugs to either treat Long-COVID or mitigate the release of inflammatory mediators from microglia. Understanding how neuro-immune and toxic triggers contribute to Long-COVID and MC on how to regulate this response, is of clinical importance (Figure 3). One of the major impediments has been the lack of appropriate disease surrogates either *in vivo* or *in vitro* (242), as well as the lack of effective inhibitors of neuroinflammation. Apparently, there have been therapeutic considerations of “stabilizing” the BBB (174, 243).

With respect to inflammation, non-steroidal anti-inflammatory drugs (NSAIDs) did not improve COVID-19 (242). Biologics have also been tried in COVID-19. Even though IL-6 has been reported to be elevated and possibly an independent risk factor, clinical trials using IL-6 inhibitors did not show any consistent benefit in COVID-19 (244). One study reported that a clinically available IL-1 antagonist significantly improved COVID-19 with secondary hemophagocytic lymphohistocytosis (sHLH) characterized by pancytopenia and hyper-coagulation (245). Glucocorticoids have been used extensively in severe, hospitalized patients with COVID-19 (246), but the results are confusing. One paper reported a reduction in mechanical ventilation and a 20 percent reduction in the mortality rate of COVID-19 patients but was also associated with longer hospital stays and longer viral clearance time (247). A systematic review and meta-analysis showed a trend toward a higher discharge rate, but the effect was minimal and not significant (246). Another analysis of 11 randomized control trials reported that systemic corticosteroids slightly reduced 30-day mortality in severe patients, but there was no benefit up to 120 days and no benefit in mild disease (248). A multicenter observational cohort study conducted in 55 Spanish intensive care units reported that early administration of high doses of dexamethasone since symptom onset could actually prove harmful for 90-day mortality (249). In fact, it has been argued that even though glucocorticosteroids may improve outcomes in severe, intubated patients with COVID-19, they could also reduce the production of antiviral IgG antibodies (250) thus hampering protection from other infections and worsening long-term outcomes (251).

Inhibition of brain inflammation could instead be accomplished with the use of some natural flavonoids (234, 252–256), but most of the evidence derives from in vitro studies. Flavonoids have been reported to prevent neuroinflammation (255–258). In particular, the flavone luteolin inhibits both microglia (259–261) and MC (262, 263), as well as related inflammatory processes (75, 255), is neuroprotective (255, 258, 264, 265), reduces cognitive dysfunction (260, 266–269), especially brain fog (29, 257, 258). Luteolin may be useful the treatment of neurodegenerative diseases (270, 271) including multiple sclerosis (272–276).

The luteolin analog, tetramethoxyluteolin (75), can inhibit secretion of the cytokines IL-1β and TNF-α (259), as well as the chemokines CCL2 and CCL5 (277) from human microglia (259, 261), and MC (172, 244). However, flavonoids are difficult to dissolve in aqueous solutions and also have poor oral absorption and bioavailability. Two formulations containing liposomal luteolin (BrainGain^®^ and FibroProtek^®^**)** were successfully used to treat a severe COVID-19 patient with brain fog (278). We have identified a novel luteolin-structurally similar flavonoid, the flavanone eriodictyol (279–281), which is partially water-soluble and may be particularly suited for development as an effective treatment because of its multiple beneficial actions (282–284), (Table 3).

**Table 3:** Beneficial actions of luteolin and eriodictyol.

• Ameliorate cognitive dysfunction
• ACE2-RBD blockers
• Anti-inflammatory
• Antioxidants
• Cardioprotective
• Hepatoprotective
• Inhibit brain injury and neurological deficits
• Inhibit synaptic dysfunction
• Inhibit oxidative stress-associated cell death
• Inhibit stress-induced deleterious effects
• Neuroprotective
• RNA polymerase inhibitors
• SARS-CoV-2 protease inhibitors

**Figure 4:**
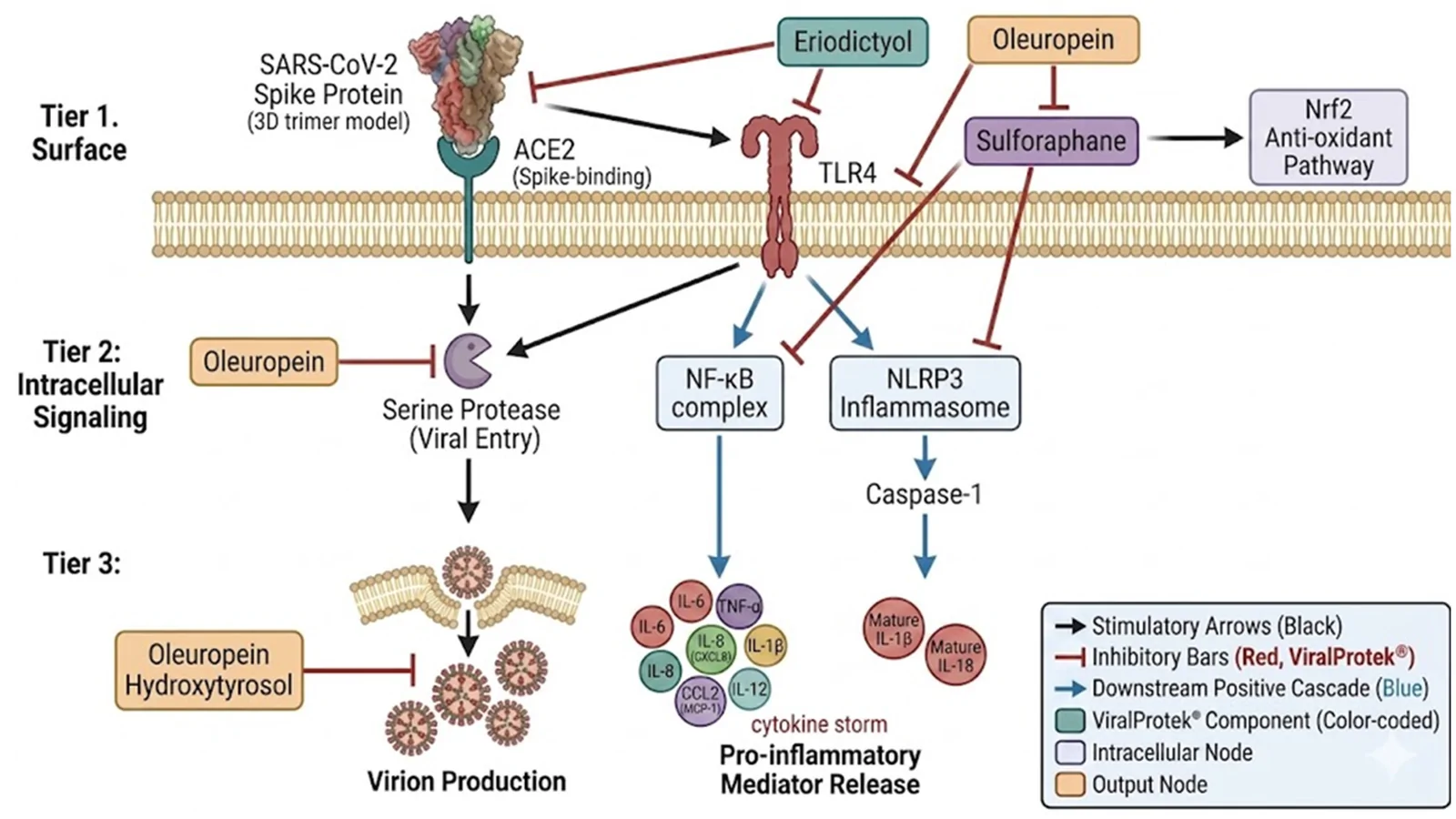
Schematic diagram showing the proposed beneficial effects of ViralProtek^®^ components on a MC or microglial cell activated by SARS-CoV-2 Spike protein. Black arrows: stimulatory pathways. Red blunt-ended bars (⊣): inhibitory actions of mainly eriodictyol, oleuropein, and sulforaphane. Blue arrows: pro-inflammatory output pathways. All components converge on NF-κB and NLRP3 signaling to reduce pro-inflammatory mediator release. Designed using Canva and Gemini-AI.

Luteolin 3′4′,5,7-tetrahydroxy-flavone) is common in celery and peppers, whereas eriodictyol 3′4′,5,7-tetrahydroxy-dihydroflavone) is mainly found in citrus fruits. Luteolin is a flavone, meaning it has a double bond between positions 2 and 3 in the C-ring, while eriodictyol is a flavanone, lacking this double bond, making it a dihydroflavone. A new, novel, dietary supplement (ViralProtek^®^) combines eriodictyol (283–286) with oleuropein from olive leaves (287–289) and sulforaphane from broccoli (290) all of which have been shown to have strong coronavirus inhibitory properties see (Figure 4). The schematic diagram illustrates a three-tiered therapeutic framework [surface, intracellular signaling (node), and output endpoints (node)] demonstrating how specific phytochemical components, focusing mainly on eriodictyol, oleuropein, and sulforaphane, interfere with viral pathogenesis and downstream immune activation. At the cellular surface (Tier 1), the model portrays the initial binding of the SARS-CoV-2 Spike protein trimer to the ACE2 receptor, alongside spike-mediated activation of TLR4. Progression through intracellular signaling pathways (Tier 2) highlights the dual activation of the NF-κB transcription complex and the NLRP3 inflammasome, which drive the enzymatic activation of Caspase-1. Finally, the terminal output phase (Tier 3) maps these parallel cascades to their specific pathological endpoints: Serine protease-mediated viral entry resulting in virion production, and the massive, uninhibited release of coordinated pro-inflammatory mediators contributing to the characteristic cytokine storm output.

### 1.11 Beneficial actions of luteolin and eriodictyol

Overall, natural flavonoids, particularly luteolin (272, 274, 275, 277) and eriodictyol (99, 280, 281) share a broad and complementary profile of pharmacological actions that make them particularly relevant to the neuroinflammatory mechanisms driving Long-COVID. Both compounds are potent inhibitors of NF-κB and NLRP3 inflammasome signaling, thereby suppressing the release of pro-inflammatory cytokines, including IL-1β, IL-6, and TNF-α, from activated microglia and MCs. Luteolin has been extensively studied in human microglial and MC models, where it additionally suppresses JNK/AP-1 and STAT1/CD40 activation pathways (265). Eriodictyol, a structurally related flavanone differing from luteolin only by the absence of the C2–C3 double bond, additionally activates the Nrf2/Keap1 antioxidant pathway, reducing ROS-mediated neuronal damage. Critically for Long-COVID, both compounds act as ACE2-RBD blockers, competitively inhibiting Spike protein binding to ACE2 and thus potentially limiting both viral entry and Spike-driven MC activation. In silico and biochemical studies further identify both as inhibitors of the SARS-CoV-2 main protease (Mpro) and RNA-dependent RNA polymerase, adding direct antiviral properties to their anti-inflammatory profile (291). In preclinical models, both are neuroprotective, reducing brain injury, inhibiting synaptic dysfunction, and ameliorating cognitive impairment, effects of direct relevance to the brain fog and memory deficits that define Long-COVID neuropsychiatric sequelae. Both compounds also exert cardioprotective and hepatoprotective effects, relevant to the multi-organ involvement seen in Long-COVID.

### 1.12 Limitations of luteolin and eriodictyol

Despite their promising preclinical profile, both luteolin and eriodictyol face significant pharmacological and clinical limitations that must be acknowledged. The most important constraint is that the overwhelming majority of evidence for their anti-neuroinflammatory and neuroprotective effects derives from in vitro cell culture studies and rodent models, with a near-complete absence of randomized controlled trial data in Long-COVID patients, a critical evidence gap that prevents firm clinical recommendations at this time. Regarding pharmacokinetics, luteolin has poor aqueous solubility and undergoes extensive hepatic first-pass glucuronidation and sulfation, resulting in low oral bioavailability and plasma concentrations that may fall below the therapeutically relevant range demonstrated in vitro (typically 5–50 μM) (292). The extent to which either compound penetrates the BBB under the neuroinflammatory conditions prevailing in Long-COVID, where BBB permeability is itself altered, remains to be quantified in dedicated studies. Additionally, at higher supplemental doses both compounds may inhibit cytochrome P450 enzymes (notably CYP3A4 and CYP1A2), raising the potential for drug–drug interactions in Long-COVID patients who frequently receive polypharmacy regimens (293). Finally, optimal dosing regimens, treatment duration, and long-term safety profiles have not been established through formal dose-finding or phase II/III clinical trials. Addressing these gaps through well-designed translational studies and placebo-controlled trials is essential before luteolin and eriodictyol or other related phytochemicals can be recommended as evidence-based therapeutic interventions for Long-COVID neuropsychiatric manifestations.

## 2 Discussion

The neuroinflammatory model proposed in this review, centered on persistent SARS-CoV-2 Spike protein driving MC and microglial activation, is supported by converging lines of evidence: direct experimental studies of Spike-driven MC activation (215, 217), emerging data on circulating Spike antigen persistence in Long-COVID (202, 203), and the reservoir framework of Proal et al. (204, 207). The epidemiological data showing that Omicron variants, despite lower overall Long-COVID prevalence, are disproportionately associated with neurological symptoms including brain fog (43), is consistent with the neuroimmune mechanisms described here, potentially reflecting differences in Spike-TLR4 interaction efficiency across variant sequences.

SARS-CoV-2 may have direct effects via its S1 protein stimulating of release of pro-inflammatory and vasoactive mediators (294) from MC and microglia that express ACE2 (73, 74). It is not yet known if the S1 protein is released extracellularly after the SARS-CoV-2 infects its target cells. Given the absence of infection of the brain discussed above, the neuropathologic findings may be due to the S1 protein. Indirect evidence of the presence of the S1 protein within the CNS is the detection of anti-S1 protein antibodies in the CSF of two children who died with COVID-19 infection and had subacute neuropsychiatric symptoms (295). Two publications reported that the S1 protein could disrupt the barrier function in an *in-vitro* model of the BBB (296) and that the S1 protein can actually cross the BBB and enter the brain in mice (297). Using transgenic mice expressing the human S1 protein, it was shown that intranasal infection of mice with SARS-CoV-2 rapidly induced ischemic-like reactivity in brain pericytes and the S1 protein reached the brain (298).

The action of the S1 protein may not be mediated only via ACE2. For instance, the S1 subunit can also bind to the surface glycoprotein neuropilin-1 (NRP-1) thus dysregulating immune responses and neuronal development (299, 300). SARS-CoV-2 can also activate TLRs, especially TLR2, leading to secretion of pro-inflammatory cytokines independent of viral entry (301, 302). Activation of TLR4 increases expression of ACE2 (303) further enhancing viral infectivity in an autocrine loop. Activation of TLRs appears to involve activation of both inflammasomes and the mammalian target of rapamycin (mTOR) (304, 305), which has been implicated in the pathogenesis of neuropsychiatric diseases (306). Elevated IL-33 receptor (sST2) has been reported in severe patients with COVID-19 infection (14). In addition, analysis of published scRNseq data from bronchoalveolar lavage fluid (extracted from patients with mild to severe COVID-19 infection) contained a population of cells that produced IL-33 and correlated with the severity of the disease (15). ^Two^ other publications reported a unique correlation of IL-12p70/IL-33 with disease severity (16) and increased expression of IL-33 in cultured epithelial cells infected with SARS-CoV-2 (17). It is therefore of interest that cultured human MC were shown to respond to IL-33 (307) by selective release of IL-43 (308).

### 2.1 Future directions

There must be better efforts to develop disease models for infection-associated conditions. Due to the complex nature of many neuropsychiatric and neurodegenerative disorders, animal models and single-cell cultures have proven inadequate to study the pathogenesis (132). This void has recently been filled by the use of cerebral organoids from human induced pluripotent stem cells (hiPSCs) derived from healthy controls and patients (133, 309–311). Human organoids are self-organized 3-D tissue cultures composed of iPSC (312–315) and have been used to investigate the pathogenesis (133, 316) of complex neurologic diseases (133, 218, 309–311, 317, 318), such Parkinson's disease (319–321) and Alzheimer's disease (322, 323). Use of human organoids has also been proposed for the study of human brain development (324, 325) and pediatric patients with neurologic diseases (311, 324), including ASD (132, 326–332). Most of these studies focused on neuronal activity (333) and neuron transcriptome status (332, 334–336). For instance, one study showed that excess neurons in embryonic-stage brain cortical organoids correlated with the severity of ASD (337). Brain organoids have also been used to study neuroinflammation (325, 338) and neuropharmacology (339). Future studies should include human organoids containing endothelial cells, MC, microglia and neurons to better represent a brain neurovascular unit.

## 3 Conclusion

This review proposes that Long-COVID neuropsychiatric and cognitive manifestations, including brain fog, fatigue, and mood disturbances, are driven by a sustained neuroinflammatory cascade initiated by SARS-CoV-2 Spike (S1) protein. We argue that Spike protein persisting within MC and macrophage ‘reservoirs,' particularly in the meninges and gut, can chronically stimulate both MC and microglia, triggering the release of pro-inflammatory, vasoactive, and neurotoxic mediators. This, in turn, disrupts the BBB, alters the HPA axis signaling, and promotes a state similar to ‘brain autoimmunity.'

The involvement of PAF and the role of platelets in COVID-19-associated coagulopathy and perivascular inflammation add a hematological dimension to the neuroinflammatory model. The paper further contextualizes these mechanisms within the broader literature on MC disorders, ASD, and neurodegenerative diseases to build a case for MC–microglia interactions as a central pathological axis.

From a therapeutic standpoint, we identify several promising avenues: natural flavonoids, particularly luteolin and the novel candidate eriodictyol, have demonstrated inhibitory effects on both microglial and MC activation in preclinical settings. The ViralProtek^®^ formulation, combining eriodictyol, oleuropein, and sulforaphane, is proposed as a multi-target nutraceutical strategy. Additionally, cerebral organoid models derived from human induced pluripotent stem cells (hiPSCs) are advocated as the most appropriate future experimental platform for studying Long COVID neuropathogenesis.

In summary, this review paper offers a conceptually rich, mechanistically grounded hypothesis for Long-COVID neurobiology that centers on MC and microglial crosstalk driven by persistent Spike protein. Rigorous clinical and translational studies including confirmation of Spike protein persistence in CNS tissues, quantification of MC and microglial activation biomarkers in Long-COVID patients, and placebo-controlled trials of the proposed nutraceutical interventions, are essential next steps before these findings can inform clinical practice.
