# Correction: Pathogen effects on the brain: the case of SARS-CoV-2 spike protein and neuro COVID

**DOI:** 10.3389/fneur.2026.1945736

**Published:** 2026-09-09

**Authors:** Theoharis C. Theoharides, Paraskevi Papadopoulou

**Affiliations:** 1 Institute for Neuro-Immune Medicine, Nova Southeastern University, Clearwater, FL, United States; 2 Department of Immunology, Tufts University School of Medicine, Boston, MA, United States; 3 Department of Natural Sciences, School of Science and Technology, Deree-The American College of Greece, Athens, Greece

**Keywords:** brain, flavonoids, inflammation, Long-COVID, Spike protein

In the 1 Introduction section third line […infection (1–4), also referred to as “Post-acute COVID” (2), “Long-COVID” or Neuro-COVID (2).] change it to […infection (1–4), also referred to as “Post-acute COVID” (2), “Long-COVID” or “Neuro-COVID” (2)]. In the 2 Discussion section, third paragraph […and correlated with the severity of the disease (15). ^*Two*^ other publications reported a unique…] change to: […and correlated with the severity of the disease (15). Two other publications reported a unique…].

There was a mistake in Figures 2 and 4. Figure 2 is actually Figure 4, and Figure 4 is Figure 2. Please switch the two Figures. Leave the captions (legends) as they appear in the published Paper.

The original version of this article has been updated.

